# HKD-CPI: high-order knowledge distillation enhanced inductive compound-protein interaction prediction

**DOI:** 10.1093/bioinformatics/btag290

**Published:** 2026-05-21

**Authors:** Zhongyu He, Xiangrong Liu, Yinghui Jiang, Junlin Xu, Yuan Lin, Shuting Jin, Leyi Wei, Youyu Wang

**Affiliations:** School of Informatics, Xiamen University, Xiamen, 361005, China; School of Informatics, Xiamen University, Xiamen, 361005, China; School of Informatics, Xiamen University, Xiamen, 361005, China; School of Computer Science and Technology, Wuhan University of Science and Technology, Wuhan, Hubei 430065, China; School of Economics, Innovation, and Technology, Kristiania University College, Bergen 5022, Norway; School of Computer Science and Technology, Wuhan University of Science and Technology, Wuhan, Hubei 430065, China; Centre for Artificial Intelligence driven Drug Discovery, Faculty of Applied Science, Macao Polytechnic University, Macao SAR 999078, China; Department of Thoracic Surgery, Sichuan Academy of Medical Sciences and Sichuan Provincial People’s Hospital, University of Electronic Science and Technology of China, Chengdu, Sichuan Province 610072, China

## Abstract

**Motivation:**

Accurately identifying compound–protein interactions (CPIs) is critical for accelerating drug discovery. Recent deep learning methods have achieved impressive results, yet they primarily focus on local structures and neighborhood information, often overlooking high-order interaction patterns shared among similar molecules.

**Results:**

In this paper, we propose HKD-CPI, a high-order knowledge-enhanced inductive framework designed to improve generalization to unseen compound–protein pairs. Specifically, HKD-CPI introduces a molecular graph tokenization mechanism that aligns compound molecular graph features with token embeddings from sequence-pretrained large language models (LLMs), effectively infusing sequence-derived semantics into structural representations. To capture shared interaction patterns among functionally similar biomolecules, we construct a hypergraph-based representation to model high-order relationships between feature-similar compound/protein groups and their binding partners. Furthermore, a knowledge distillation strategy is further adopted to transfer high-order interaction knowledge from the hypergraph to a lightweight student model, enabling efficient and robust CPI prediction. Extensive experiments demonstrate that HKD-CPI outperforms existing state-of-the-art methods in inductive CPI prediction tasks. In particular, it achieves an average improvement of 4.94% in AUROC and 3.64% in AUPRC over the best-performing baseline across five benchmark datasets.

**Availability and implementation:**

Our code and data are available at https://github.com/Hezy618/HKD-CPI.

## 1 Introduction

Identifying compound–protein interactions (CPIs) is a fundamental step in drug discovery (Du *et al.* 2022). However, the experimental validation of CPIs is typically time-consuming and expensive, which renders large-scale screening infeasible (Zhang *et al.* 2024). To address this limitation, computational approaches have been developed to predict potential CPIs by learning interaction patterns from large-scale known data, providing a more efficient and cost-effective alternative for drug development (Yin *et al.* 2024). Among these, deep learning methods have gained considerable attention due to their strong capability in capturing complex molecular features (Xiang *et al.* 2024).

Nevertheless, existing deep learning models typically rely heavily on interaction patterns observed in the training data, which limits their generalization ability to unseen compounds and proteins (Xia *et al.* 2024). This limitation has motivated researchers to explore more flexible and generalizable solutions. With the remarkable success of large language models (LLMs) in modeling sequential data, recent studies have attempted to leverage pre-trained LLMs to extract informative representations from the sequence data of compounds and proteins (Murakumo *et al.* 2023). By leveraging knowledge from large-scale training corpora, LLMs can infer features of unseen compounds and proteins from their sequences, effectively mitigating the cold-start problem in CPI prediction (Hua *et al.* 2025). To further exploit multimodal molecular information, a number of approaches have extended pre-trained LLMs to incorporate both sequence-based and graph-based representations of compounds (Xia *et al.* 2024). While these methods commonly extract features separately from each modality followed by fusion, their focus on modality-specific information often comes at the cost of overlooking deeper multimodal correlations and complementarity, ultimately constraining the effectiveness of multimodal feature representation.

In addition to modeling the individual features of compounds and proteins, their structural similarity during binding encodes valuable patterns that can facilitate more accurate CPI prediction (Trudeau *et al.* 2023). For instance, SgCPI (Xia *et al.* 2024) identifies structurally similar visible proteins for each unseen protein during training and uses them as intermediates to retrieve two-hop compound neighbors, which are then considered potential binding candidates. Experimental evidence shows that structurally similar proteins tend to share common binding compounds. However, in large-scale CPI prediction scenarios, explicitly computing structural similarity across massive compound and protein datasets is computationally expensive (Wu *et al.* 2024). Moreover, high-quality, experimentally verified structural data are not always available (Hadipour *et al.* 2025), significantly limiting the practicality and scalability of such approaches in real-world drug screening tasks.

**Figure 1:**
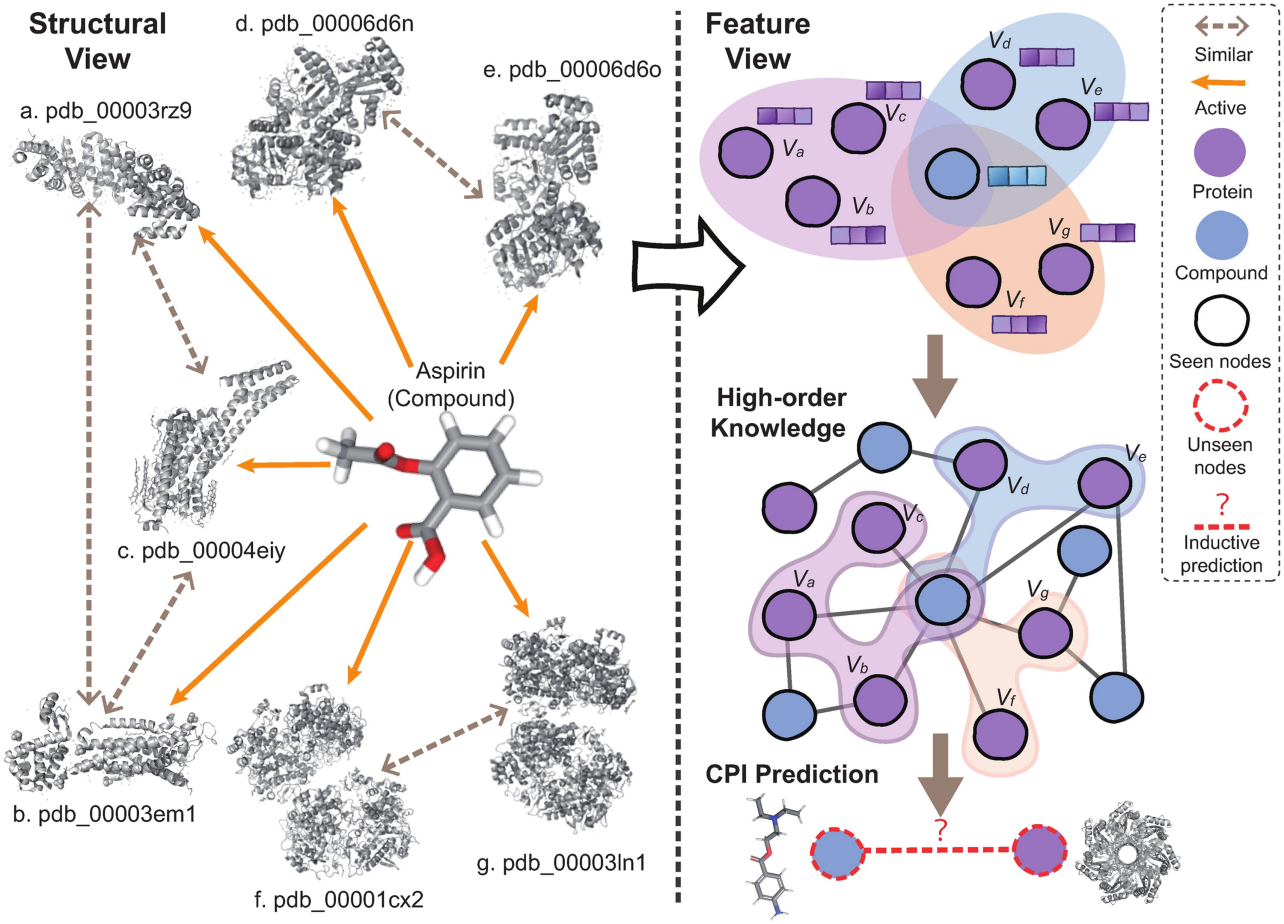
An example illustrating the transition from structure-based to feature-based similarity between compounds and proteins during binding.

To address the aforementioned challenges, we propose HKD-CPI, a novel inductive framework for CPI prediction enhanced by high-order knowledge distillation. Specifically, we enhance the molecular graph features of compounds by associating them with token embeddings derived from sequence-pretrained LLMs. This allows the model to transfer informative patterns from known sequences to improve the representation of molecular graphs. Furthermore, we reformulate similarity relationships in the representation space. Instead of relying on explicit chemical structures, we compute feature-level similarity based on learned representations of compounds and proteins, enabling a more efficient and flexible modeling of interaction patterns. Importantly, the resulting n-ary relationships, which are formed between groups of feature-similar compounds or proteins and their shared binding partners, cannot be naturally captured by traditional graph structures. To this end, we construct a compound–protein hypergraph based on molecular feature similarity, leveraging the expressive power of hypergraphs to model high-order interactions where each hyperedge can connect multiple related entities. This enables a shift from structure-based similarity to a feature-based perspective, as illustrated in Fig. 1. Finally, we adopt a knowledge distillation strategy to transfer the high-order interaction knowledge encoded in the hypergraph into a lightweight student model, thereby improving its inductive prediction performance on unseen compound–protein pairs.

In summary, the main contributions of this paper are summarized as follows:

- We propose a novel HKD-CPI framework that performs high-order knowledge distillation to improve inductive CPI prediction.
- We design a graph tokenizer module that extracts molecular features and associates them with token embeddings from a sequence-pretrained LLM, leveraging the LLM’s sequence information to enhance the graph representations of compounds.
- We construct a hypergraph based on feature similarity to encode high-order interaction information, and transfer this knowledge to a student model via distillation, leading to more effective generalization in out-of-distribution scenarios.
- Experimental results show that our method achieves significantly superior performance on five benchmark datasets, and case studies further demonstrate its effectiveness in identifying potential CPIs.

## 2 Preliminaries & problem definition

### 2.1 Hypergraph representation learning

A hypergraph can be represented by the formula $G_{H}=(V,E)$, where $V=\{v_{1},\ldots ,v_{\left|V\right|}\}$ denotes the set of nodes, and $E=\{e_{1},\ldots ,e_{\left|E\right|}\}$ denotes the set of hyperedges. Each hyperedge can connect an arbitrary number of nodes and can be viewed as a non-empty set of nodes. During computation, the hypergraph structure is conveyed through the adjacency matrix $H\in {\left\{0,1\right\}}^{\left|V|\times |E\right|}$, where $H_{i,j}=1$ indicates that node *i* is connected to hyperedge *j*. The goal of hypergraph representation learning is to learn the node feature representation $X_{v}\in R^{|V|\times d_{1}}$ and the hyperedge feature representation $X_{e}\in R^{|E|\times d_{2}}$ based on the hypergraph $G_{H}$, where the feature dimensions are $d_{1}$ and $d_{2}$, respectively.

### 2.2 Inductive CPI prediction

Inductive CPI prediction refers to the challenging setting where both the compound and the protein in the test pair are entirely unseen during training. Formally, given a known interaction graph $G_{\text{CPI}}=\{C,P,E_{\text{CPI}}\}$, where $C=\{c_{1},\ldots ,c_{\left|C\right|}\}$ represents the set of compounds, $P=\{p_{1},\ldots ,p_{\left|P\right|}\}$ represents the set of proteins, and $E_{\text{CPI}}=\{(c,p)|c\in C,p\in P\}$ represents the known active interactions between compounds and proteins. The goal is to learn the interaction patterns between compounds and proteins from the known data $G_{\text{CPI}}$, which enables the extraction of effective compound features $X_{c}$ and protein features $X_{p}$. These features are used to predict whether an unseen interaction pair $(c,p)\notin E_{\text{CPI}}$ is active or inactive. In the inductive setting, the unknown interaction pair must satisfy c∉C and p∉P, and the task is to predict the activity of this new interaction based on the learned patterns from the known data.

## 3 Methodology

**Figure 2:**
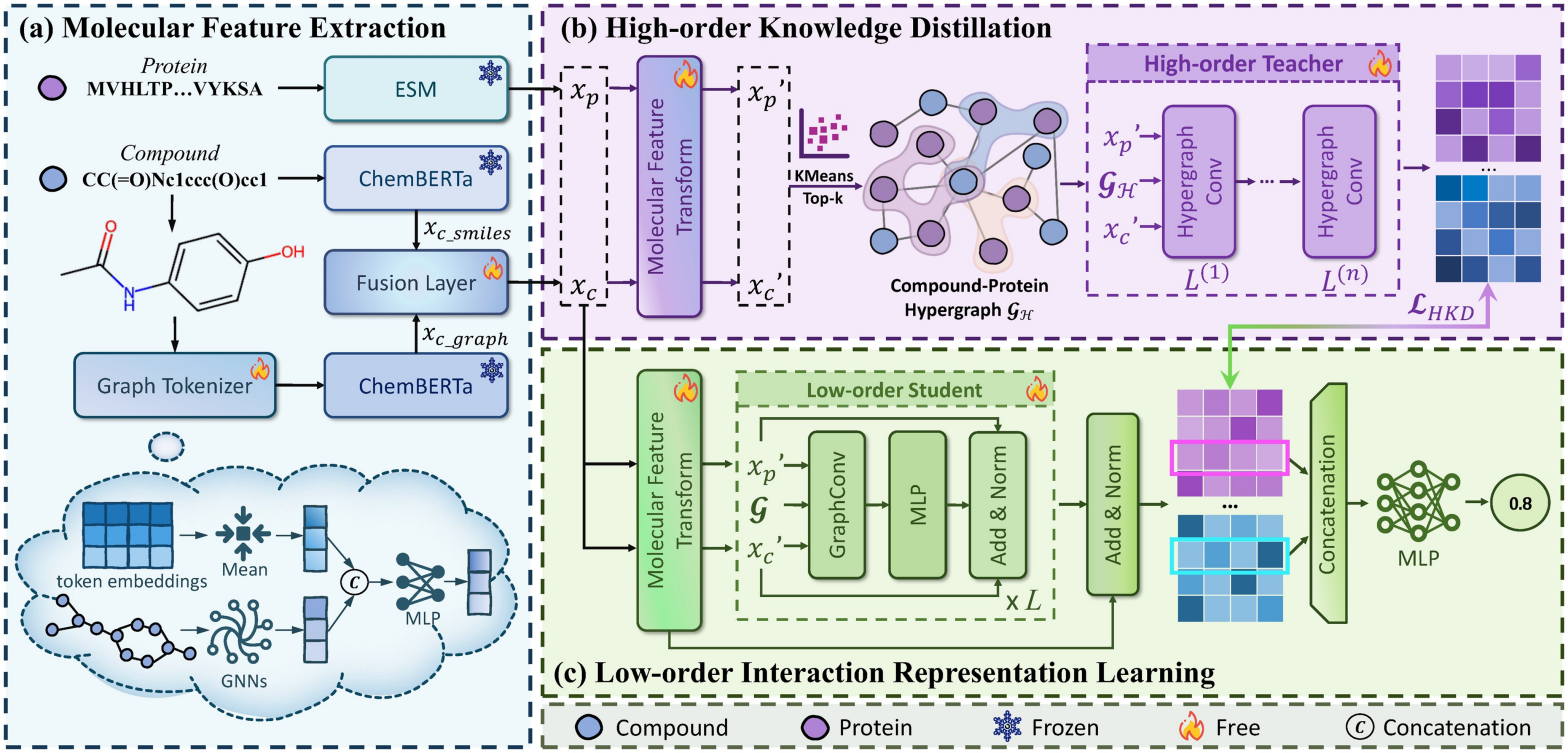
Framework of HKD-CPI. (a) Molecular Feature Extraction: Extracts sequence features of compounds and proteins using a pretrained LLM, then enhances compound molecular graph features via a Graph Tokenizer module. (b) High-order Knowledge Distillation: Models high-order interactions among molecular feature-similar compound/protein groups via hypergraph construction, transferring knowledge learned by the hypergraph convolutional encoder to the student model through distillation. (c) Low-order Interaction Representation Learning: Learns compound-protein interaction patterns from known CPI networks by integrating distilled high-order interactions from (b), then performs CPI prediction.

The proposed HKD-CPI consists of three modules: Molecular Feature Extraction, High-order Knowledge Distillation, and Low-order Interaction Representation Learning. The detailed computational flow is shown in Fig. 2.

### 3.1 Molecular feature extraction

To leverage known data for obtaining effective initial feature representations of compounds and proteins, we design a molecular feature extraction module based on pre-trained biological LLMs. This module extracts molecular features from the input SMILES sequences of compounds and the amino acid sequences of proteins. Specifically, we use ChemBERTa (Chithrananda *et al.* 2020) and ESM (Rives *et al.* 2021) to extract features from the input sequence information. However, biological LLMs trained solely on sequence data lack structural context, which is essential for accurately capturing the chemical semantics of small molecules. To address this issue, inspired by Graph2Token (Wang *et al.* 2024), we design a tokenization module for the compound molecular graph. This module associates features learned from molecular graphs by GNNs with token embeddings from sequence-pretrained LLMs, allowing for further enhancement of the molecular graph features based on sequence information. The computational flow of the molecular graph encoder and the molecular graph feature tokenization is formalized as:


(1a) $$X_{a}^{\left(l+1\right)}=\text{MLP}(\text{LN}(\sigma (\text{GCN}(X_{a}^{\left(l\right)},G_{c}^{\mathrm{mol}}))+X_{a}^{\left(l\right)}))$$

(1b) $$x_{c\_\mathrm{graph}}=W(\frac{1}{m}\sum_{i=1}^{m}X_{a,i})+b$$

(1c) $$x_{c\_\mathrm{token}}=\text{MLP}(x_{c\_\mathrm{graph}}∥\frac{1}{n}\sum_{i=1}^{n}X_{\mathrm{token},i})$$

where *n* is the number of compounds, *m* is the number of atoms within compound *c*, $X_{a}$ is the feature matrix of atomic nodes in the molecular graph $G_{c}^{\mathrm{mol}}$ of compound *c*, and $X_{\mathrm{token}}$ is the sequence token embedding matrix of the compound. $X_{a,i}$ and $X_{\mathrm{token},i}$ denote the feature vector of the *i*-th atom and the *i*-th compound’s sequence token embedding, respectively. W is a learnable parameter, and b is the bias term. LN denotes the LayerNorm operation, and MLP is a 2-layer multi-layer perceptron. ∥ indicates concatenation.

To ensure the consistency of the feature space and dimensions of compounds and proteins in subsequent modules, while integrating molecular features from different modalities, we design a molecular feature transformation module, as formulated below:


(2a) $$x_{c}=\text{MLP}(x_{c\_\mathrm{smiles}}∥x_{c\_\mathrm{graph}})$$

(2b) $$x_{c}^{\prime }=\text{MLP}(\text{LN}((Wx_{c}+b)+x_{c}))$$

where $x_{c}$ denotes the compound feature obtained by fusing two modalities, $x_{c}^{\prime }$ represents the transformed compound feature. $x_{c\_\mathrm{smiles}}$ denotes the sequence-based representation of the compound extracted by ChemBERTa, W is a learnable transformation matrix, and *b* represents the bias term.

For the molecular feature transformation of proteins, we perform molecular feature transformation on the amino acid sequence features output by the pre-trained LLM, as formulated below:


(3) $$x_{p}^{\prime }=\text{MLP}(\text{LN}((Wx_{p}+b)+x_{p}))$$

where $x_{p}$ represents the protein features obtained from the pre-trained LLM, while $x_{p}^{\prime }$ denotes the transformed protein features. W is a learnable transformation matrix, and b is the bias term.

### 3.2 High-order knowledge distillation

To model the n-ary relationships between groups of feature-similar compounds or proteins and their shared binding partners, we construct a compound-protein hypergraph based on feature similarity. The high-order interaction patterns between compounds and proteins are then extracted using a hypergraph convolution encoder. Knowledge distillation is employed to transfer the learned high-order knowledge to the student model, thereby enhancing its inductive CPI prediction performance. Specifically, we first perform clustering of compounds/proteins based on their transformed molecular features using KMeans, dividing them into $k_{1}$ clusters. For each compound/protein, we sample its nearest protein/compound cluster as potential high-order neighbors. During hyperedge sampling, we select the top $k_{2}$ high-order neighbor nodes based on cosine similarity between compound and protein features to form hyperedges. Specifically, we construct two sub-hypergraphs, $H_{c}$ and $H_{p}$, centered on compounds and proteins, respectively. The hyperedges in $H_{c}$ have the form $\left\{c,p_{1},\ldots ,p_{k_{2}}\right\}$, while the hyperedges in $H_{p}$ take the form $\left\{p,c_{1},\ldots ,c_{k_{2}}\right\}$. To unify the node indexing of compounds and proteins, we add $\mathrm{len}(X_{c})$ to the protein indices. Finally, the two hypergraph structures are merged. The process of hypergraph construction is detailed in Algorithm 1. To extract high-order interaction information from the constructed hypergraph, we use HGNN (Feng *et al.* 2019) as the teacher model for feature extraction, as formulated below:


(4) $$X^{\left(l+1\right)}=\sigma \left(D_{v}^{-1/2}HWD_{e}^{-1}H^{⊤}D_{v}^{-1/2}X^{\left(l\right)}\Theta^{\left(l\right)}\right)$$

where $D_{v}$ and $D_{e}$ are the degree matrices for the nodes and hyperedges, respectively, Θ is a learnable weight, W is a learnable weight matrix, and σ is a nonlinear activation function. X is the node feature matrix obtained by concatenating the feature matrices of compounds and proteins.

Subsequently, to achieve feature-level knowledge distillation, we compute a similarity loss between the teacher model and the student model based on features learned from high-order and low-order structural representations, as formulated below:


(5) $$L_{\text{HKD}}=-\frac{1}{N}\sum_{i=1}^{N}\;\text{log}\;\left(\frac{\;\text{exp}\;\left(\frac{X_{i}^{\text{tea}}\cdot X_{i}^{\text{stu}}}{\tau }\right)}{\sum_{j=1}^{N}\;\text{exp}\;\left(\frac{X_{i}^{\text{tea}}\cdot X_{j}^{\text{stu}}}{\tau }\right)}\right)$$

where $X^{\text{tea}}$ and $X^{\text{stu}}$ represent the feature representations of compounds and proteins learned by the teacher model and the student model, respectively, τ is the temperature coefficient, and *N* represents the total number of compound and protein nodes.

### 3.3 Low-order interaction representation learning

To capture interaction patterns between compounds and proteins from known data, we construct the CPI network as a compound-protein bipartite graph and perform interaction pattern learning using GNNs. Specifically, the transformed molecular features serve as the initial node features for compounds and proteins. We then apply a GNN model to update the feature representations by incorporating the topological structure of the bipartite graph. To address the issue of excessive smoothing in GCN, we introduce a two-layer MLP after each GCN layer and use residual connections to merge the node features of compounds and proteins from the previous layer, as formulated below:


(6) $$X^{\left(l+1\right)}=\text{LN}(\text{MLP}(\text{GCN}(X^{\left(l\right)},G_{\text{CPI}}))+X^{\left(l\right)})$$

where $G_{\text{CPI}}$ represents the bipartite graph structure formed by the known CPI data, and X represents the node matrix obtained by concatenating the feature matrices of compounds and proteins.

Algorithm 1 Compound-Protein Hypergraph Construction.

**Input**: Compound features $X_{c}$; protein features $X_{p}$; number of clusters $k_{1}$; hyperedge sampling degree $k_{2}$

**Output**: Hypergraph *H*

**Function**: K-Means clustering *kMeans*; cosine distance function *dis*; K smallest distance index selection *topK*;

1: **function**  Construct_H($X_{\mathrm{query}},X_{\mathrm{ref}},k_{1},k_{2}$)

2:  $C\leftarrow \mathrm{kMeans}(X_{\mathrm{ref}},k_{1})$

3:  Initialize hypergraph *H*

4:  **for** *i* in $\mathrm{range}(\mathrm{len}(X_{\mathrm{query}}))$  **do** 

5:    H[i].insert(i)

6:    $D_{\mathrm{center}}\leftarrow \mathrm{dis}(C.\mathrm{center},X_{\mathrm{query}}[i])$

7:    $\mathrm{cluster}\_\mathrm{idx}\leftarrow \mathrm{topK}(D_{\mathrm{center}},1)$

8:    ref_list←C[cluster_idx]

9:    $D_{qr}\leftarrow \mathrm{dis}(X_{\mathrm{query}}[i],X_{\mathrm{ref}}[\mathrm{ref}\_\mathrm{list}])$

10:    $\mathrm{sampled}\leftarrow \mathrm{topK}(D_{qr},k_{2})$

11:    **for** *j* in *sampled* **do** 

12:     H[i].insert(ref_list[j])

13:    **end for** 

14:  **end for** 

15:  **return** *H*

16: **end function** 

17: *H_c_*  Construct_H (**X**_c_, **X***_p_*, *k*_1_, *k*_2_)

18: *H_p_*  Construct_H (**X***_p_*, **X***_c_*, *k*_1_, *k*_2_)

19: **return**  $H_{c}+H_{p}$

Before making predictions, we first introduce the initially input transformed compound and protein features in the form of residuals. Next, for the unknown CPI pair (c,p), we concatenate their feature vectors and use an MLP for interaction prediction, as formulated below:


(7) $$y=\text{MLP}(\text{LN}(x_{cs}+x_{c}^{\prime })∥\text{LN}(x_{ps}+x_{p}^{\prime }))$$

where *y* denotes the model’s prediction of whether compound *c* interacts with protein *p*, and $x_{c}^{\prime }$ and $x_{p}^{\prime }$ represent the transformed molecular features of the compound and protein, respectively. $x_{cs}$ and $x_{ps}$ correspond to the node features of the compound and protein output by the student model.

### 3.4 Training and optimization

For model training, high-order knowledge distillation and CPI prediction serve as the optimization objectives. The high-order knowledge distillation loss, $L_{\text{HKD}}$, is computed as shown in Equation (5), while binary cross-entropy loss is used for the CPI prediction task in the inductive setting, as formulated below:


(8) $$L_{\text{BCE}}=-\frac{1}{N}\sum_{i=1}^{N}\left[y_{i}\;\text{log}(p_{i})+(1-y_{i})\;\text{log}(1-p_{i})\right]$$

where *N* is the number of samples, $y_{i}$ is the label of the *i*-th sample, and $p_{i}$ is the model’s prediction.

The total loss consists of the high-order knowledge distillation loss and the binary cross-entropy loss for CPI prediction, as formulated below:


(9) $$L=L_{\text{BCE}}+\lambda L_{\text{HKD}}$$

where λ denotes the weight of $L_{\text{HKD}}$, which is set to 1. The rationale and experimental results regarding this choice are provided in Supplementary Appendix E.

Additionally, we adopt an early stopping strategy for model training and use AdamW (Loshchilov and Hutter 2019) to optimize the parameters of all involved modules.

## 4 Experiments

### 4.1 Experimental setups

#### 4.1.1 Datasets

CPI prediction experiments were conducted under an inductive setting using five datasets: BindingDB (Gilson *et al.* 2016), BioSNAP (Zitnik *et al.* 2018), KIBA (Tang *et al.* 2014), C.elegans (Liu *et al.* 2015), and PDBbind 2016 (Cang *et al.* 2018), with dataset statistics provided in Table 1. Following the data split scheme proposed by GraphBAN (Hadipour *et al.* 2025), we ensured that compounds and proteins in the test data were unseen during training. Specifically, we clustered the features of compounds and proteins within each dataset, then randomly selected 60% of the clusters, along with their corresponding CPIs, for training. The remaining 40% of the clusters were split into validation and test sets in a 4:1 ratio. Detailed information on the datasets is provided in Supplementary Appendix A.

**Table 1:** Statistics of the datasets used in the experiments.

Dataset	|C|	|P|	Active	Inactive
BindingDB	14643	2623	20,674	28,525
BioSNAP	4505	2181	13,830	13,634
KIBA	2068	229	22,729	95,525
C.elegans	1767	1876	26,659	3,893
PDBbind 2016	3227	2410	4,053	4,053

#### 4.1.2 Baselines and evaluation strategies

To objectively evaluate the performance of the proposed method, comparisons are made with several classic and recent models for inductive CPI prediction. The models utilized include: GraphDTA (Nguyen *et al.* 2021), GraphsformerCPI (Ma *et al.* 2023), DrugBAN (Bai *et al.* 2023), FusionDTI (Meng *et al.* 2025), and GraphBAN (Hadipour *et al.* 2025). AUROC and AUPRC are employed as evaluation metrics. The average results of the inductive CPI prediction experiments, conducted with five random runs and corresponding standard deviations for each dataset, are compared with those of other baseline models.

#### 4.1.3 Implementation details

We conduct our experiments on NVIDIA RTX 4090 GPUs with 24GB of RAM, using Python 3.9.11 and PyTorch 1.12.1 to ensure a consistent hardware and software environment. For the inductive CPI prediction experiments, we set the number of clusters for constructing the compound-protein hypergraph to 25, with the hyperedge degree set to 3. We use the Deep Hypergraph library (https://github.com/iMoonLab/DeepHypergraph) to build a two-layer HGNN (Feng *et al.* 2019) as the high-order teacher model. The number of layers in the GNNs of the Graph Tokenizer is set to 2. The complete hyperparameter configurations are listed in Supplementary Appendix B.

### 4.2 Main results

We conduct inductive CPI prediction experiments using five datasets from GraphBAN (Hadipour *et al.* 2025). For each dataset, five runs with random initialization are performed, with average results reported in Table 2. Experimental results demonstrate that HKD-CPI achieves optimal performance on the majority of the three metrics across all five datasets. Notably, under the inductive setting on PDBbind 2016, which is characterized by relatively limited known data and greater experimental difficulty, HKD-CPI maintains strong performance, achieving a 13.04% improvement in AUROC over the second-best method. These results establish HKD-CPI as a robust state-of-the-art solution for inductive CPI prediction, particularly excelling in data-scarce scenarios.

**Table 2:** Performance comparison of various models for CPI prediction under inductive settings.

Dataset	Metric	GraphsformerCPI	GraphDTA	DrugBAN	FusionDTI	GraphBAN	HKD-CPI(ours)
BioSNAP	AUROC	0.620${\;}_{\pm 0.026}$	0.694${\;}_{\pm 0.012}$	0.687${\;}_{\pm 0.029}$	0.672${\;}_{\pm 0.021}$	0.751 ${\;}_{\pm 0.023}$	**0.816** ${\;}_{\pm 0.006}$
	AUPRC	0.598${\;}_{\pm 0.026}$	0.728${\;}_{\pm 0.008}$	0.748${\;}_{\pm 0.032}$	0.687${\;}_{\pm 0.021}$	0.807 ${\;}_{\pm 0.018}$	**0.847** ${\;}_{\pm 0.010}$
BindingDB	AUROC	0.552${\;}_{\pm 0.008}$	0.541${\;}_{\pm 0.025}$	0.586${\;}_{\pm 0.043}$	0.514${\;}_{\pm 0.043}$	0.618 ${\;}_{\pm 0.036}$	**0.626** ${\;}_{\pm 0.035}$
	AUPRC	0.460${\;}_{\pm 0.044}$	0.453${\;}_{\pm 0.031}$	0.489${\;}_{\pm 0.073}$	0.425${\;}_{\pm 0.021}$	**0.532** ${\;}_{\pm 0.056}$	0.527 ${\;}_{\pm 0.027}$
KIBA	AUROC	0.633${\;}_{\pm 0.006}$	0.631${\;}_{\pm 0.042}$	0.633${\;}_{\pm 0.019}$	0.582${\;}_{\pm 0.008}$	**0.654** ${\;}_{\pm 0.017}$	0.652 ${\;}_{\pm 0.009}$
	AUPRC	0.379 ${\;}_{\pm 0.014}$	0.314${\;}_{\pm 0.070}$	0.275${\;}_{\pm 0.055}$	0.249${\;}_{\pm 0.030}$	0.306${\;}_{\pm 0.041}$	**0.380** ${\;}_{\pm 0.011}$
C.elegans	AUROC	0.635${\;}_{\pm 0.025}$	0.789${\;}_{\pm 0.050}$	0.868${\;}_{\pm 0.022}$	0.716${\;}_{\pm 0.072}$	0.892 ${\;}_{\pm 0.022}$	**0.910** ${\;}_{\pm 0.037}$
	AUPRC	0.606${\;}_{\pm 0.031}$	0.718${\;}_{\pm 0.135}$	0.819${\;}_{\pm 0.059}$	0.708${\;}_{\pm 0.089}$	0.854 ${\;}_{\pm 0.058}$	**0.887** ${\;}_{\pm 0.042}$
PDBbind 2016	AUROC	0.523${\;}_{\pm 0.069}$	0.567${\;}_{\pm 0.030}$	0.557${\;}_{\pm 0.043}$	0.598 ${\;}_{\pm 0.062}$	0.561${\;}_{\pm 0.055}$	**0.676** ${\;}_{\pm 0.057}$
	AUPRC	0.531${\;}_{\pm 0.063}$	0.556${\;}_{\pm 0.038}$	0.566${\;}_{\pm 0.024}$	0.597 ${\;}_{\pm 0.026}$	0.560${\;}_{\pm 0.042}$	**0.657** ${\;}_{\pm 0.070}$

**Bold** values indicate best performance while underlined values show second-best results.

### 4.3 Hyperparameter tuning analysis

**Figure 3:**
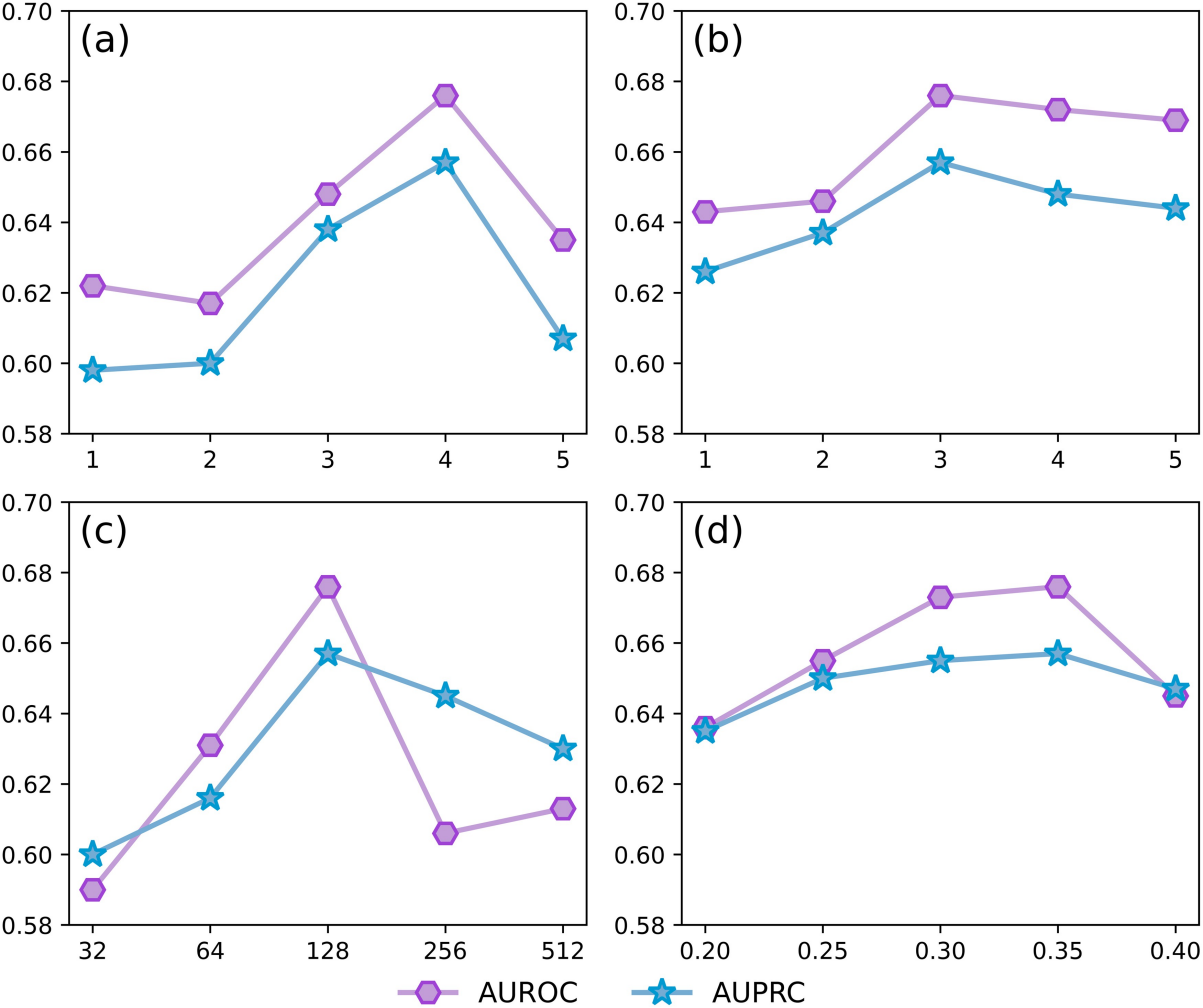
Visualization of model performance under different hyperparameter settings on the PDBbind 2016 dataset.

To assess the impact of hyperparameters on model performance, we conduct experiments on the PDBbind 2016 dataset, varying four key hyperparameters: (1) hyperedge sampling degree, (2) embedding dimension, (3) number of layers, and (4) dropout rate. Each parameter is tested across five values: {1, 2, 3, 4, 5} for layers and sampling degree, {32, 64, 128, 256, 512} for embedding dimension, and {0.2, 0.25, 0.3, 0.35, 0.4} for dropout rate, while other parameters are held constant according to the main experiment. The comprehensive results are presented in Fig. 3. Panels (a), (b), and (c) demonstrate that model performance improves with an increase in the number of layers, hyperedge sampling degree, and embedding dimension, achieving optimal results with 4 layers, a sampling degree of 3, and an embedding dimension of 128. Further increases in these parameters lead to a decline in performance, suggesting that while appropriate values expand the model’s capacity, excessive values lead to over-smoothing and overfitting. An optimal hyperedge sampling degree allows the model to effectively explore high-order interactions between compounds and proteins, whereas higher values introduce noise, reducing the amount of useful information. Panel (d) shows that both AUROC and AUPRC achieve their optimal performance at a dropout rate of 0.35. Further increases in the dropout rate cause performance degradation, indicating that an optimal dropout rate prevents overfitting and enhances performance, while higher rates lead to over-regularization, diminishing the model’s learning ability. Additionally, we assess the impact of different high-order teacher models and hypergraph clustering partition numbers on HKD-CPI, with the results presented in Supplementary Appendix C.

### 4.4 Ablation study

To evaluate the impact of different components of HKD-CPI on overall performance, we conduct experiments on the BioSNAP and PDBbind 2016 datasets, maintaining the same parameter configuration as in the main experiment. Specifically, we assess the effects of High-order Knowledge Distillation (HKD), Molecular Graph Features, and Graph Tokenizer on model performance. For the **HKD** component, we remove the teacher model and related loss, relying solely on the student model for CPI prediction. For **Molecular Graph Features**, we replace the molecular graph features with the sequence features of compounds in subsequent computations. For **Graph Tokenizer**, we only use the molecular graph encoder to obtain molecular graph features, omitting tokenization and subsequent feature enhancement.

Table 3 presents the experimental results. The first three sets show that using only pre-trained sequence features for inductive CPI prediction yields the worst performance. Introducing molecular graph features and the Graph Tokenizer improves the feature representation by integrating multimodal data, resulting in an average AUROC improvement of 5.93% across two datasets compared to using sequence features alone. The last three sets demonstrate that incorporating HKD into the corresponding settings from the first three groups leads to further improvements in AUROC, with average gains of 7.54%, 5.82%, and 7.07% across the two datasets. These results highlight that the high-order knowledge provided by HKD enhances the model’s generalization to unseen compounds and proteins. Notably, HKD outperforms the inclusion of additional modality features in boosting model performance. Combining all three components, HKD-CPI achieves the best performance.

**Table 3:** Ablation study results of HKD-CPI on BioSNAP and PDBbind 2016 datasets.

HKD	Molecular Graph	Graph Tokenizer	**BioSNAP**		**PDBbind 2016**	
			AUROC	AUPRC	AUROC	AUPRC
×	×	×	0.737${\;}_{\pm 0.014}$	0.782${\;}_{\pm 0.017}$	0.582${\;}_{\pm 0.067}$	0.573${\;}_{\pm 0.056}$
×	✓	×	0.752${\;}_{\pm 0.011}$	0.787${\;}_{\pm 0.010}$	0.614${\;}_{\pm 0.049}$	0.593${\;}_{\pm 0.064}$
×	✓	✓	0.775${\;}_{\pm 0.009}$	0.801${\;}_{\pm 0.013}$	0.621${\;}_{\pm 0.027}$	0.613${\;}_{\pm 0.035}$
✓	×	×	0.781${\;}_{\pm 0.012}$	0.815 ${\;}_{\pm 0.011}$	0.635${\;}_{\pm 0.058}$	0.619${\;}_{\pm 0.043}$
✓	✓	×	0.793 ${\;}_{\pm 0.015}$	0.811${\;}_{\pm 0.010}$	0.652 ${\;}_{\pm 0.033}$	0.644 ${\;}_{\pm 0.061}$
✓	✓	✓	**0.816** ${\;}_{\pm 0.006}$	**0.847** ${\;}_{\pm 0.010}$	**0.676** ${\;}_{\pm 0.057}$	**0.657** ${\;}_{\pm 0.070}$

**Figure 4:**
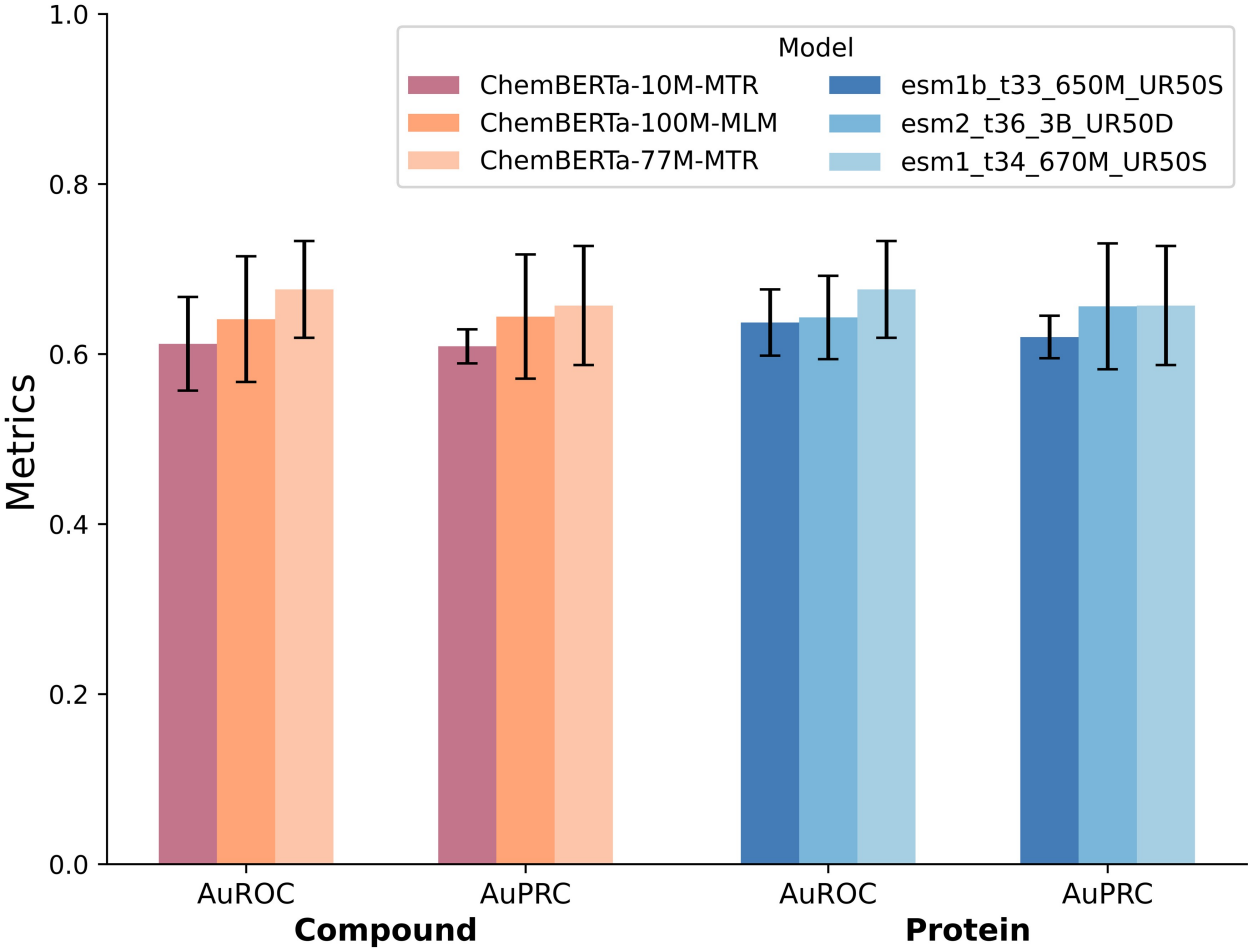
Performance comparison of ChemBERTa and ESM variants with varying parameter scales on the PDBbind 2016 dataset.

Furthermore, we investigate the influence of parameter scale on predictive performance. Specifically, we conduct evaluation experiments on the PDBbind 2016 dataset to compare various configurations of ChemBERTa and ESM series. The selected variants include {ChemBERTa-10M-MTR, ChemBERTa-100M-MLM, ChemBERTa-77M-MTR} and {esm2_t36_3B_UR50D, esm1b_t33_650M_UR50S, esm1_t34_670M_UR50S}, with all other hyperparameters kept consistent with the main experiments. As illustrated in Fig. 4, the ChemBERTa-77M-MTR variant consistently outperforms its counterparts across all metrics, achieving a 5.46% improvement in AUROC compared to the larger ChemBERTa-100M-MLM. Similarly, for the ESM series, esm1_t34_670M_UR50S achieves the optimal performance, surpassing the much larger esm2_t36_3B_UR50D by 5.13% in terms of AUROC. These empirical results suggest that model performance is not strictly monotonic with respect to parameter scale. On one hand, undersized models are hindered by a representational bottleneck due to limited capacity, which restricts their ability to capture complex biomolecular interactions. On the other hand, increasing parameter scale enhances expressive power but may introduce feature redundancy and incur prohibitive computational overhead without commensurate performance gains. Consequently, striking a balance between effectiveness and efficiency, we employ ChemBERTa-77M-MTR and esm1_t34_670M_UR50S as the default backbone models for our framework.

### 4.5 Case study

**Figure 5:**
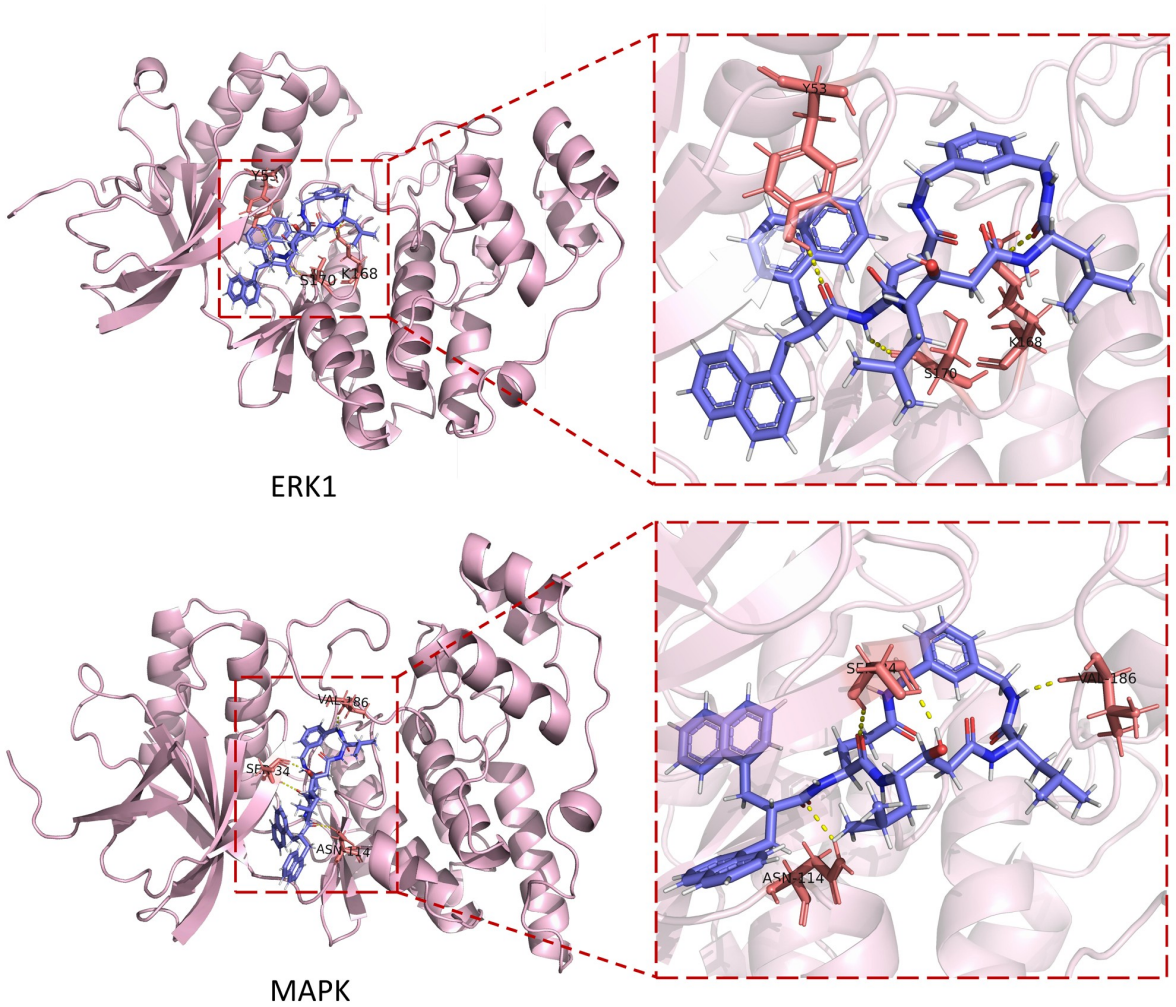
Visualization of CPI prediction for structurally similar proteins ERK1 and MAPK, and their common binding compound, Compound 1 [with SMILES CC(C)C[C@@H]1NC(=O)CC@HC@HNC(=O)C@@HCCC(=O)NCc2cccc (c2)CNC1 = O].

To assess the application of HKD-CPI in real-world drug discovery scenarios and the effectiveness of the strategy that reconstructs the similarity relationship between compounds and proteins from a feature perspective, we selected chemically similar unseen proteins, Mitogen-Activated Protein Kinase (MAPK) and Extracellular Signal-Regulated Kinase 1 (ERK1), for an inductive CPI prediction case study. Specifically, we first trained the model on the PDBbind 2016 dataset, using compounds from the test data as candidate binding compounds for these two protein samples. The trained model was then used to perform CPI prediction scoring. Subsequently, we selected the top-ranked common binding compound from the CPI prediction results of both proteins and conducted molecular docking experiments. The docking results are shown in Fig. 5. The results clearly show that Compound 1 is the common binding compound predicted by HKD-CPI for the two structurally similar proteins from the same candidate compounds. This compound effectively binds to both proteins, confirming the validity of HKD-CPI’s strategy of reformulating similarity relationships in feature space to enhance the model’s generalization capability. Furthermore, it demonstrates the effectiveness of HKD-CPI in identifying compound candidates that may interact beneficially with the target proteins.

## 5 Conclusion

In this paper, we introduce HKD-CPI, a novel approach that enhances inductive CPI prediction performance through high-order knowledge distillation. Specifically, HKD-CPI integrates molecular graph features of compounds with token embeddings derived from sequence-pretrained LLMs, thereby enriching the compound’s structural representation with sequence-based information. To uncover shared interaction patterns among functionally similar biomolecules, HKD-CPI reformulates similarity relationships in the representation space, employing a hypergraph to model n-ary relationships between compounds, proteins, and their binders based on molecular feature similarity. Through knowledge distillation, the high-order interaction patterns learned by the teacher model from the hypergraph are transferred to the student model, further enhancing its inductive CPI prediction capability. Extensive experimental results demonstrate that HKD-CPI significantly outperforms state-of-the-art methods in inductive CPI prediction tasks. Future work will focus on exploring additional perspectives to deepen our understanding of compound-protein interactions, leveraging AI to provide innovative and powerful tools for drug discovery.

## Supplementary Material

btag290_Supplementary_Data

## Data Availability

The data and code underlying this article are available in a public GitHub repository at: https://github.com/Hezy618/HKD-CPI.
