# Supplementary material for: HKD-CPI: high-order knowledge distillation enhanced inductive compound-protein interaction prediction
Source: Bioinformatics. 2026 May 21;42(6):btag290. doi: 10.1093/bioinformatics/btag290 (PMC13224967; doi:10.1093/bioinformatics/btag290)
Supplement: btag290_Supplementary_Data [file btag290_supplementary_data.zip › 25-May-2026_014439_Appendix_final.pdf]

A Dataset Details

In this section, we provide a detailed description of the datasets employed in the primary experiments of this study. To assess the model's performance in predicting Compound-Protein Interactions (CPIs) within an inductive setting, we perform clustering on both compounds and proteins based on their respective molecular features. The clustering results are then leveraged to partition the CPI dataset into training, validation, and test sets, ensuring that no compounds or proteins from the test set appear in the training phase. Specifically, we adopt the clustering strategy and settings from GraphBAN, utilizing the extended connectivity fingerprint with a depth of 4 (ECFP4) for compounds and the 3-mer frequency feature for proteins. To measure similarity relationships between molecular features, we compute both cosine and Jaccard distances, which are subsequently used for clustering. The compound and protein clusters are randomly sampled in a 3:2 ratio to form the training set, while the remaining clusters, along with the associated CPIs, are divided into validation and test sets in a 4:1 ratio. The detailed statistical data of the datasets are provided in Table 1.

Table 1. Statistics of the datasets used in the experiments.

**Dataset**	$\lvert\mathcal{C}\rvert$	$\lvert\mathcal{P}\rvert$	**Active**	**Inactive**	**Compound Clusters**	**Protein Clusters**
BindingDB	14643	2623	20,674	28,525	2780	1693
BioSNAP	4505	2181	13,830	13,634	2387	1978
KIBA	2068	229	22,729	95,525	666	195
C.elegans	1767	1876	26,659	3,893	934	1793
PDBbind 2016	3227	2410	4,053	4,053	1407	1119

B Hyperparameter Settings

This section provides complete hyperparameter configurations corresponding to the dataset-specific results reported in Table 2 of our main experiments for HKD-CPI. The detailed settings are documented in Table 2.

Table 2. Hyperparameter settings for HKD-CPI across different datasets.

**Hyperparameter**	**BioSNAP**	**BindingDB**	**KIBA**	**C.elegans**	**PDBbind 2016**
Number of Layers (teacher)	2	2	2	2	2
Number of Layers (student)	3	4	2	4	4
Number of Layers (predictor MLP)	2	2	2	2	2
Embedding Dim	256	256	256	256	128
Number of Clusters k_1	25	25	25	25	25
Hyperedge Degree k_2	3	3	3	3	3
Learning Rate	1e-4	1e-4	1e-4	1e-4	1e-4
Weight decay	1e-5	1e-5	1e-5	1e-5	1e-5
Dropout Rate	0.25	0.25	0.2	0.25	0.35
Early Stopping Patience	55	55	55	55	55

C Additional Experimental Results

Table 3. Performance comparison of HKD-CPI with different high-order teacher models for inductive CPI prediction.

**HyperGCN**	**UniGCN**	**DHCF**	**HGNN+**	**HGNN**	**BioSNAP**		**PDBbind 2016**	
					**AUROC**	**AUPRC**	**AUROC**	**AUPRC**
✓					$0.792_{\pm 0.035}$	$0.826_{\pm 0.027}$	$0.636_{\pm 0.051}$	$0.627_{\pm 0.053}$
	✓				$0.794_{\pm 0.021}$	$0.819_{\pm 0.018}$	$0.647_{\pm 0.049}$	$0.635_{\pm 0.049}$
		✓			$0.786_{\pm 0.027}$	$0.823_{\pm 0.022}$	$0.643_{\pm 0.051}$	$0.639_{\pm 0.066}$
			✓		$\underline{0.807}_{\pm 0.015}$	$\underline{0.841}_{\pm 0.011}$	$\underline{0.664}_{\pm 0.064}$	$\underline{0.649}_{\pm 0.052}$
				✓	$\mathbf{0.816}_{\pm 0.006}$	$\mathbf{0.847}_{\pm 0.010}$	$\mathbf{0.676}_{\pm 0.057}$	$\mathbf{0.657}_{\pm 0.070}$

C.1 Variants of High-order Teacher Models

We investigate the impact of different high-order teacher models on the overall performance of HKD-CPI. Specifically, we implement HyperGCN(Yadati et al., 2019), DHCF (Ji et al., 2020), UniGCN (Huang and Yang, 2021), and HGNN+ (Gao et al., 2023) using the Deep Hypergraph library[1]. To control for confounding variables, we maintain identical experimental configurations to those in our main experiments, replacing only the high-order teacher models in the High-order Knowledge Distillation module, while keeping all architectures as two-layer models. As shown in Table 3, both HGNN and HGNN+ demonstrate marginally superior performance as high-order teachers in HKD-CPI's distillation module under identical configurations. Notably, compared to HGNN+, HGNN exhibits more stable average performance across all five datasets, with a particularly significant 1.2% AUROC improvement on the PDBbind 2016 dataset—a challenging benchmark due to its relatively limited known data for inductive CPI prediction.

C.2 Impact of Cluster Partition Counts

We further investigated the impact of varying the number of clusters on the performance of HKD-CPI during the construction of the compound-protein hypergraph. Specifically, we kept all other hyperparameters consistent with the main experiment, while changing only the value of the number of clusters k_1 from the set $\{20, 25, 30, 35, 40\}$. We then observed how the performance metrics of HKD-CPI on PDBbind 2016 varied with these different values of k_1. As shown in the experimental results presented in Figure 1, when the number of clusters was set to 25, the similarity relationships at the feature level between compounds and proteins were more concentrated. This led to hyperedges sampled from the resulting clusters that contained more effective high-order interaction information. However, increasing the number of clusters further led to a dispersion of similarity relationship information, which reduced the amount of effective information in the constructed hypergraph, thus weakening the generalization capability of HKD-CPI.

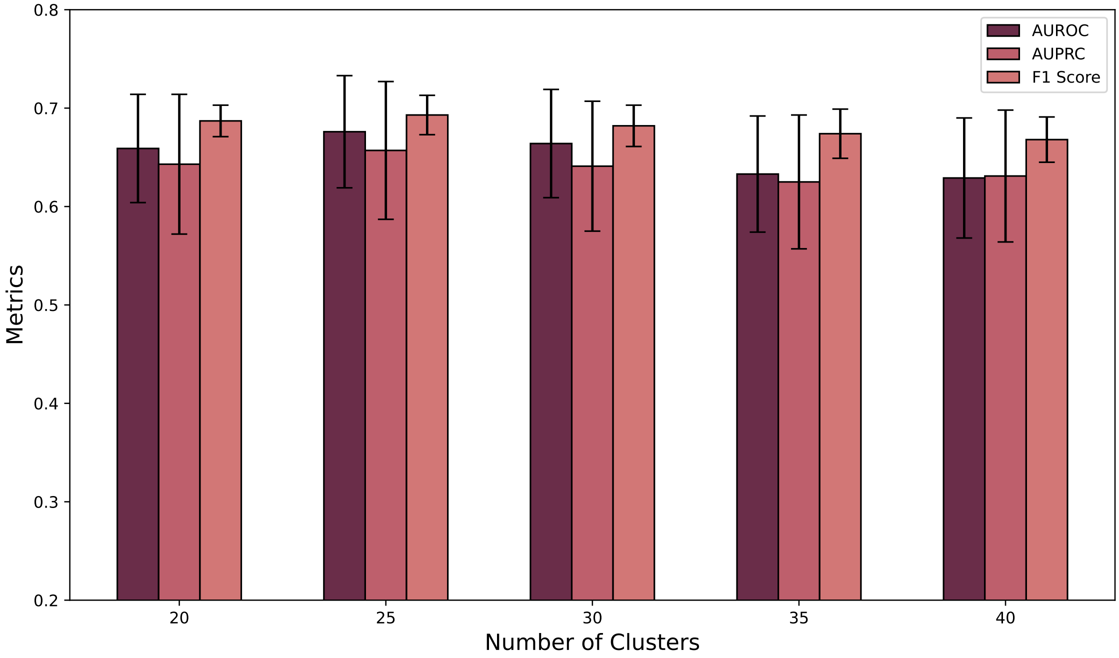

Fig. 1: Performance comparison of HKD-CPI with different hypergraph cluster partitioning numbers for inductive CPI prediction.

C.3 Additional CPI Case Studies

In addition to the example results presented in the main text, we conducted an additional CPI prediction case study using another set of chemically similar and unseen proteins. Specifically, we selected the Beta-adrenergic receptor (BAR) and Dopamine Receptor 2 (D2) as protein samples, and trained HKD-CPI on the relatively small PDBbind 2016 dataset. All unseen compounds from the test data were used as candidate binding compounds for both proteins to predict potential CPIs. We selected Compound 2, which ranked highest in the prediction results and was a common binding compound for both protein samples, as the final prediction, followed by molecular docking experiments. The visualization results, as shown in Figure 2, reveal that Compound 2, predicted by HKD-CPI from the PDBbind 2016 test data, effectively binds to both proteins A and B, which are chemically similar. These additional case study results further demonstrate that HKD-CPI can predict common and effective binding compounds for chemically similar proteins, even with limited

[1] `https://github.com/iMoonLab/DeepHypergraph`

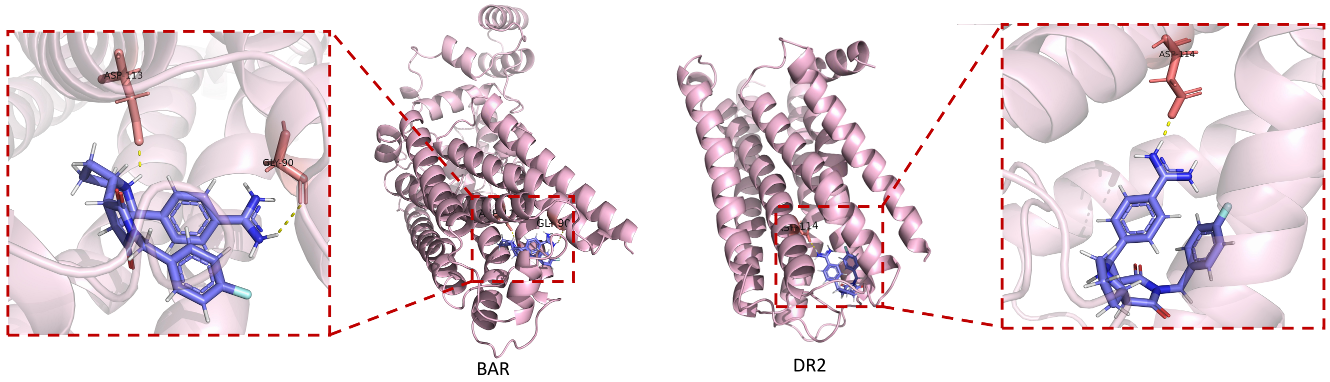

Fig. 2: Visualization of CPI prediction for structurally similar proteins BAR and DR2, and their common binding compound, Compound 2 (with SMILES NC(=[NH2+])c1ccc([C@H]2[C@H]3C(=O)N(Cc4ccc(F)cc4)C(=O)[C@H]3[C@@H]3CCC[N@@H+]32)cc1).

Table 4. Performance comparison of the model with different values of the weight λ of $\mathcal{L}_{HKD}$.

0.6	0.7	0.8	0.9	1.0	BioSNAP		PDBbind 2016	
					AUROC	AUPRC	AUROC	AUPRC
✓					$0.810_{\pm0.014}$	$\underline{0.843}_{\pm0.014}$	$0.653_{\pm0.065}$	$0.626_{\pm0.066}$
	✓				$0.809_{\pm0.014}$	$0.842_{\pm0.013}$	$0.647_{\pm0.049}$	$0.635_{\pm0.049}$
		✓			$0.811_{\pm0.013}$	$0.843_{\pm0.011}$	$0.653_{\pm0.044}$	$0.650_{\pm0.043}$
			✓		$\underline{0.814}_{\pm0.013}$	$0.841_{\pm0.014}$	$\underline{0.657}_{\pm0.64}$	$\underline{0.654}_{\pm0.66}$
				✓	$\mathbf{0.816}_{\pm0.006}$	$\mathbf{0.847}_{\pm0.010}$	$\mathbf{0.676}_{\pm0.057}$	$\mathbf{0.657}_{\pm0.070}$

known data, by leveraging high-order interaction patterns between compounds and proteins discovered through feature similarity in the high-order knowledge distillation module. This further supports the model's potential for practical applications in drug development.

D Related Works

LLM-based CPI prediction.

LLM-based methods leverage pre-trained models to extract feature representations from compound and protein sequence or structural data for subsequent computations and CPI predictions. For example, SgCPI-KD(Xia et al., 2024) uses GROVER(Rong et al., 2020) to extract molecular graph features from compounds and employs MSA Transformer(Rao et al., 2021) and GraSR(Xia et al., 2022) to extract and fuse protein sequence and structural features. ChemGLaM(Koyama et al., 2025) utilizes MolFormer(Ross et al., 2022) and ESM-2(Lin et al., 2023) for molecular feature extraction from compound and protein sequences, respectively, and applies an attention mechanism for CPI prediction. GraphBAN(Hadipour et al., 2025) extracts sequence features from compounds and proteins using ChemBERTa(Chithrananda et al., 2020) and ESM(Rives et al., 2021), then combines these with molecular graph features via Graph Neural Networks (GNNs) and protein sequence features via Convolutional Neural Networks, fusing them with pre-trained molecular features. However, most existing approaches process multi-modal data in isolation and fuse them only at a late stage, overlooking the intrinsic correlations and complementary semantics between different modalities.

Knowledge distillation in CPI prediction.

Knowledge distillation, an effective model compression and knowledge transfer technique, has been widely applied across various domains, including CPI prediction. SgCPI-KD (Xia et al., 2024) uses the node features learned by a teacher model to sample potential neighbor nodes for unseen compounds and proteins, addressing the cold-start problem in inductive CPI. FedKD-CPI (Wang et al., 2025) combines federated learning and knowledge distillation to achieve multi-institutional collaboration in CPI prediction while ensuring data privacy. GraphBAN (Hadipour et al., 2025) designs an overall framework based on a knowledge distillation strategy, where the teacher model focuses on network structural information from known data, while the student model focuses on node attributes, thus improving the accuracy of inductive CPI prediction. However, these approaches predominantly focus on transferring pairwise or domain-specific representations and overlook high-order n-ary interaction patterns between feature-similar biomolecules and their binding partners, which are essential for robust generalization to unseen compounds and proteins.

E The Effect of the Weight λ

We further investigate the effect of different values of the weight of $\mathcal{L}_{HKD}$ on model training performance on the BioSNAP and PDBbind 2016 datasets. Specifically, we set $\lambda \in \{0.6, 0.7, 0.8, 0.9, 1.0\}$ while keeping all other settings identical to the main experiments. As shown in Table 4, the model is not particularly sensitive to the choice of λ; however, on both datasets, the best performance is achieved at $\lambda = 1.0$, and decreasing the weight further leads to a noticeable drop in performance. In particular, on the more challenging PDBbind 2016 dataset, the performance variation is more pronounced, where the optimal value $\lambda = 1.0$ yields a relative improvement of 4.48% in AUROC compared to $\lambda = 0.7$.

F Effect of Soft-Label-Based Distillation Loss

We further introduced a soft-label-based loss into the distillation process to investigate the impact of this training strategy on the overall predictive performance of the model. Specifically, we computed the Kullback–Leibler (KL) divergence between the teacher model's soft label predictions and the student model's outputs, using it as the soft-label-based distillation loss $\mathcal{L}_{\text{KL}}$, which shares the same weighting coefficient λ as $\mathcal{L}_{\text{HKD}}$. Experiments were conducted on the BioSNAP and PDBbind 2016 datasets, with all other settings consistent with those of the main experiments.

As shown in Table 5, the best performance is achieved when only $\mathcal{L}_{\text{HKD}}$ is applied, yielding an average AUROC improvement of 3.85% and 1.97% across the two datasets compared to using $\mathcal{L}_{\text{KL}}$ alone and combining both losses, respectively. Although incorporating the soft-label-based loss enables the student model to more accurately mimic the teacher model's prediction process and outcomes, it also constrains the student's ability to explore the complex compound–protein interactions, leading to a slight decline in overall performance.

Table 5. Performance comparison of the model with and without the soft-label-based distillation loss on the BioSNAP and PDBbind 2016 datasets.

$\mathcal{L}_{\text{KL}}$	$\mathcal{L}_{\text{HKD}}$	BioSNAP		PDBbind 2016	
		AUROC	AUPRC	AUROC	AUPRC
✓		$\underline{0.806}_{\pm 0.015}$	$\underline{0.842}_{\pm 0.013}$	$0.635_{\pm 0.041}$	$\underline{0.635}_{\pm 0.053}$
	✓	$\mathbf{0.816}_{\pm 0.006}$	$\mathbf{0.847}_{\pm 0.010}$	$\mathbf{0.676}_{\pm 0.057}$	$\mathbf{0.657}_{\pm 0.070}$
✓	✓	$0.805_{\pm 0.013}$	$0.842_{\pm 0.013}$	$\underline{0.659}_{\pm 0.046}$	$0.633_{\pm 0.051}$

G The Impact of Adopting More Complex Feature Fusion Strategies

We further investigate the impact of incorporating a more sophisticated cross-attention mechanism to integrate the compound's sequence and molecular graph representations on the overall model performance. Specifically, we alternately treat the sequence and graph features as the query (Q), while using the other modality's features as the key (K) and value (V), to compute bidirectional cross-attention between the two modalities. The outputs from both directions are summed and averaged to replace the original `Concat + MLP` fusion strategy. The experimental results are summarized in Table 6. As shown, employing a more complex cross-attention mechanism yields only a 2.74% improvement in AUPRC on the PDBbind 2016 dataset compared to the `Concat + MLP` strategy. However, the latter achieves 5.78% higher AUROC on both datasets. This suggests that, for the current task, a simple linear fusion method is sufficient to capture the main complementary information between the two modalities, whereas an overly complex cross-attention mechanism may introduce additional parameters and noise, thereby compromising the model's generalization ability.

Table 6. Performance comparison on the BioSNAP and PDBbind 2016 datasets under different compound feature fusion strategies.

Fusion Strategy	BioSNAP		PDBbind 2016	
	AUROC	AUPRC	AUROC	AUPRC
**Cross Attention**	$\underline{0.792}_{\pm 0.008}$	$\underline{0.832}_{\pm 0.013}$	$\underline{0.625}_{\pm 0.041}$	$\mathbf{0.675}_{\pm 0.038}$
**Concat + MLP**	$\mathbf{0.816}_{\pm 0.006}$	$\mathbf{0.847}_{\pm 0.010}$	$\mathbf{0.676}_{\pm 0.057}$	$\underline{0.657}_{\pm 0.070}$

References

S. Chithrananda, G. Grand, and B. Ramsundar. Chemberta: Large scale self-supervised pretraining for molecular property prediction. *ArXiv*, 2020. doi: 10.48550/arXiv.2010.09885. URL `https://doi.org/10.48550/arXiv.2010.09885`.

Y. Gao, Y. Feng, S. Ji, and R. Ji. Hgnn+: General hypergraph neural networks. *IEEE Transactions on Pattern Analysis and Machine Intelligence*, 45(3):3181–3199, 2023. doi: 10.1109/TPAMI.2022.3182052.

H. Hadipour, Y. Li, Y. Sun, et al. Graphban: An inductive graph-based approach for enhanced prediction of compound-protein interactions. *Nature Communications*, 16:2541, 2025. doi: 10.1038/s41467-025-57536-9. URL https://doi.org/10.1038/s41467-025-57536-9.

J. Huang and J. Yang. Unignn: a unified framework for graph and hypergraph neural networks. In Z.-H. Zhou, editor, *Proceedings of the Thirtieth International Joint Conference on Artificial Intelligence, IJCAI-21*, pages 2563–2569. International Joint Conferences on Artificial Intelligence Organization, 8 2021. doi: 10.24963/ijcai.2021/353. URL https://doi.org/10.24963/ijcai.2021/353. Main Track.

S. Ji, Y. Feng, R. Ji, X. Zhao, W. Tang, and Y. Gao. Dual channel hypergraph collaborative filtering. In *Proceedings of the 26th ACM SIGKDD International Conference on Knowledge Discovery & Data Mining*, KDD '20, page 2020–2029, New York, NY, USA, 2020. Association for Computing Machinery. ISBN 9781450379984. doi: 10.1145/3394486.3403253. URL https://doi.org/10.1145/3394486.3403253.

T. Koyama, H. Tsumura, R. Okita, K. Yamazaki, A. Hasegawa, K. Imamura, T. Kato, H. Iwata, R. Kojima, H. Inoue, S. Matsumoto, and Y. Okuno. Chemical genomics language model toward reliable and explainable compound-protein interaction exploration. *bioRxiv*, 2025. doi: 10.1101/2024.02.13.580100. URL https://www.biorxiv.org/content/early/2025/04/06/2024.02.13.580100.

Z. Lin, H. Akin, R. Rao, B. Hie, Z. Zhu, W. Lu, N. Smetanin, R. Verkuil, O. Kabeli, Y. Shmueli, A. dos Santos Costa, M. Fazel-Zarandi, T. Sercu, S. Candido, and A. Rives. Evolutionary-scale prediction of atomic-level protein structure with a language model. *Science*, 379(6637):1123–1130, 2023. doi: 10.1126/science.ade2574. URL https://www.science.org/doi/abs/10.1126/science.ade2574.

R. M. Rao, J. Liu, R. Verkuil, J. Meier, J. Canny, P. Abbeel, T. Sercu, and A. Rives. Msa transformer. In *Proceedings of the 38th International Conference on Machine Learning*, pages 8844–8856. PMLR, 2021.

A. Rives et al. Biological structure and function emerge from scaling unsupervised learning to 250 million protein sequences. *Proc. Natl Acad. Sci. USA*, 118(e2016239118), 2021. doi: 10.1073/pnas.2016239118.

Y. Rong, Y. Bian, T. Xu, W. Xie, Y. Wei, W. Huang, and J. Huang. Self-supervised graph transformer on large-scale molecular data. *Advances in Neural Information Processing Systems*, 33:12559–12571, 2020.

J. Ross, B. Belgodere, V. Chenthamarakshan, I. Padhi, Y. Mroueh, and P. Das. Large-scale chemical language representations capture molecular structure and properties. *Nature Machine Intelligence*, 4(12):1256–1264, 2022. doi: 10.1038/s42256-022-00580-7.

X. Wang, Q. Zhao, and J. Wang. Fedkd-cpi: Combining the federated knowledge distillation technique to accomplish synergistic compound-protein interaction prediction. *Methods*, 234:275–283, 2025. ISSN 1046-2023. doi: https://doi.org/10.1016/j.ymeth.2024.12.014. URL https://www.sciencedirect.com/science/article/pii/S1046202325000076.

C. Xia, S.-H. Feng, Y. Xia, X. Pan, and H.-B. Shen. Fast protein structure comparison through effective representation learning with contrastive graph neural networks. *PLoS Computational Biology*, 18(7):e1009986, 2022. doi: 10.1371/journal.pcbi.1009986. URL https://doi.org/10.1371/journal.pcbi.1009986.

Y. Xia, X. Pan, and H.-B. Shen. Heterogeneous sampled subgraph neural networks with knowledge distillation to enhance double-blind compound-protein interaction prediction. *Structure*, 32(5):611–620.e4, 2024. ISSN 0969-2126. doi: 10.1016/j.str.2024.02.004. URL https://doi.org/10.1016/j.str.2024.02.004.

N. Yadati, M. Nimishakavi, P. Yadav, V. Nitin, A. Louis, and P. Talukdar. Hypergcn: A new method for training graph convolutional networks on hypergraphs. In H. Wallach, H. Larochelle, A. Beygelzimer, F. d'Alché-Buc, E. Fox, and R. Garnett, editors, *Advances in Neural Information Processing Systems*, volume 32. Curran Associates, Inc., 2019. URL https://proceedings.neurips.cc/paper_files/paper/2019/file/1efa39bcaec6f3900149160693694536-Paper.pdf.
